# Transcriptomics and the hunt for Disease X; A view point from Ebola and COVID-19 outbreaks

**DOI:** 10.1016/j.amsu.2022.104552

**Published:** 2022-09-03

**Authors:** Amna Siddiqui, Alishba Adnan

**Affiliations:** Department of MBBS, Karachi Medical and Dental College, 74700, Pakistan

**Keywords:** Transcriptome, Genome, COVID-19, Disease X, Ebola virus

Respected Editor

WHO has listed several priority diseases, including Disease X, with epidemic potential for minimal medical countermeasures. Disease X represents Pathogen X, an infectious agent known as ‘an unknown etiologic agent’ of a future outbreak with epidemic or pandemic potential. The introduction of the term Disease X aims to prepare public healthcare systems worldwide before it becomes a threat. This will reduce the time lag between identifying causes of outbreaks and approving vaccines/treatments to curb them from turning into "the next pandemic." In addition to the Personal View by Simpson S. et al. [1], discusses the rapid development of antidotes for Pathogen X, which might become the next public health emergency. We aim to briefly share our stance on the significance of comparing transcriptomic profiles of previous and existing outbreaks as analyzed by Alsamman M. et al. [2]. Therefore, the outlook would cover how the Ebola virus can aid in tackling the existing COVID-19. Similarly, transcriptomic analyses can enhance knowledge of the pathogenesis of Disease X. Thus, more information may help us combat such an inevitability efficiently.

As aforementioned, transcriptome analysis helps to perceive all the RNA transcripts of an organism and its transcriptome. All this information is recorded in the DNA while the RNA serves as an intermediate molecule in transcription. It enables us to grip the changes in gene expression across different organisms like viruses [3]. Therefore, following the update of March’20, the Project based on the comparison of host responses to Ebola Virus Disease (EVD) gathered crucial information about COVID-19 by applying transcriptome analysis for the Ebola project to enhance understanding of the pathogenesis and immune responses to novel SARS-COV-2 [4]. Additionally, Guinea's just-ended outbreak showed that the genomic sequence found behind the virus was similar to that identified in the 2014–2016 outbreak. However, more studies are needed to fill the knowledge gap for a link between the two outbreaks [5]. Eventually providing aid in understanding the pace of mutations of COVID-19. Moving forward, there is an urgent need for new antiviral strategies, a recent discovery uncovered that expression of p41 in cells blocked the virus's cathepsin-dependent entry pathway. Which in turn, interferes with an early stage of viral entry that host cells commonly used to protect against diverse viruses, including those that cause Ebola and COVID-19 [6].

Moreover, the analysis by Alsamman M. et al. [2]compared five transcriptomic profiles for cell host infection with SARS-CoV-2, EBOV, H1N1, MERS-CoV, and SARS-CoV. The study identifies several critical aspects of the host response to SARS-CoV-2 infection and outlines the relationship between EBOV's cellular host response and SARS-CoV-2, where many genes and GO terms are enriched. This could include essential immunity genes and biological pathways that can be used to understand the pathogenesis of SARS-CoV-2 infection. Similarly, by comparing transcriptomic profiles of different viruses as implicit by the study of Alsamman M. et al. [2], we can enhance our understanding of the mechanisms governing the changes in gene expression that underlie disease outbreaks [[4], [5], [6]] and eventually would ease our hunt for Disease X.

## Sources of funding

None to declare.

## Ethical approval

None to declare.

## Consent

None to declare.

## Author contribution

All authors have contributed equally to the manuscript.

## Registration of research studies

1. Name of the registry:
2. Unique Identifying number or registration ID:
3. Hyperlink to your specific registration (must be publicly accessible and will be checked):

## Guarantor

None to declare.

## Declaration of competing interest

None to declare.
